# Division of Myocardial Enzyme Reference Intervals in Population Aged 1 to <18 Years Old Based on Fisher's Optimal Segmentation Method

**DOI:** 10.1155/2020/2013148

**Published:** 2020-03-31

**Authors:** Wenjia Guo, Qi Zhou, Yanan Jia, Jiancheng Xu

**Affiliations:** ^1^Department of Laboratory Medicine, First Hospital of Jilin University, Changchun 130021, China; ^2^Department of Pediatrics, First Hospital of Jilin University, Changchun 130021, China; ^3^Department of Laboratory Medicine, Shanxi Bethune Hospital, Taiyuan 030032, China

## Abstract

**Background:**

Reference interval (RI) research is to make it a concise, effective, and practical diagnostic tool. This study aimed to establish sex- and age-specific RI for myocardial enzyme activity in population aged 1–<18 years old in Changchun, China.

**Methods:**

Healthy subjects (*n* = 6,322, 1–<18 years old) were recruited from communities and schools. Aspartate aminotransferase (AST), lactate dehydrogenase (LDH), creatine kinase (CK), and creatine kinase isoenzyme (CKMB) were measured using an automatic biochemical analyzer. Fisher's optimal segmentation method was used to partition by including percentiles as impact factors, aiming at minimizing the sum of the squares of the total dispersion into groups as splitting sequence of ordered data.

**Results:**

AST decreased gradually and was partitioned as 1, 2∼<10 and 10∼<18 years old. LDH presented disparate descending rate among 1∼<4, 4∼<12, and 12∼<18 years old. CK stood quite stable with the same RI in all ages. CKMB began to differ at 6 years of age sexually and then remained stable during 6∼<14 years old for male while it continued to decline in female. Cardiac development was partitioned as 1∼<6, 6∼<13, and 13∼<18 years old using multiple percentiles from massive data that reflect characteristics of totality as impact factors.

**Conclusions:**

Fisher's optimal segmentation method excelled for multidimensionality, continuity, and loop calculating as dealing with RIs for myocardial enzymes activity and cardiac development process despite limitations. In future, impact of partition on the overall interval should be delved into.

## 1. Introduction

Reference interval (RI) is currently a hot spot in laboratory medicine. It is defined as the range between 2.5th percentile and 97.5th percentile of a certain indicator in healthy population [1]. As an important part of clinical laboratory and modern medicine, RI could provide valuable information for patients about diagnosis, progression, treatment, and outcome [2].

Studies have shown that RI is related to ethnicity [3], environment [4], diet [5], and others. Therefore, the establishment work must be carefully determined, taking into account the potential impact factors [6], such as enrolled individuals [7] and methodology [8]. Many research groups such as CALIPER (Canadian Laboratory Initiative in Pediatric Reference Intervals) [9] and KiGGS (German Health Interview and Examination Survey for Children and Adolescents) [10] have made great progress in this field [11].

RI partition is to divide the indicator into several stages according to the changes and distinct differences during growth and development, providing reliable and practical information for effective supervision, prevention, and diagnosis as well as management of public health. At present, the commonly applied partitioning method is to determine the changing point visually, and then use *Z* test, nonparametric method, or robust method to prove classification statistically based on actual data. Thus, the partition points are subjectively decided [12]. There could be too many groupings leading to inconvenience for clinical application, or the grouping is so little that they weaken the rationality.

We consider that partition problem be regarded as the cluster analysis of multidimensional ordered series, that is, the partition point with the largest difference between groups is found in massive data. Fisher's optimal segmentation is a method for clustering ordered samples, showing characteristics of multifactor, continuous time series and the best classification by loop calculation. This study was to investigate myocardial enzymes partition: aspartate aminotransferase (AST), lactate dehydrogenase (LDH), creatine kinase (CK), and creatine kinase isoenzyme (CKMB) in healthy children and adolescents aged 1–<18 years old from Jilin Province, China and establish a RI of cardiac development combining the 4 indicators into the model.

## 2. Material and Methods

Fisher's optimal segmentation, as cluster analysis of ordered samples, is based on the sum of the squares of the total deviations of the classifications, achieving minimal internal differences and maximum differences among groups.

Using *B*(*n*,  *k*), for representing *n* ordered samples {*x*1,  *x*2,…, *Xn*} into *k* classes, this segmentation can be expressed as follows:

(1) $$\begin{array}{c} P1=\left\{i_{1},\;i_{1}+1\ldots \;i_{2}-1\right\},\\ P2=\left\{i_{2},\;i_{2}+1\ldots \;i_{3}-1\right\},\\ \vdots \\ Pk=\left\{i_{k},\;i_{k}+1,\ldots ,n\right\}. \end{array}$$

The subpoint is 1=*i*_1_ < *i*_2_ < …< *i*_*k*_ < *n*=*i*_*k*+1_ − 1(that is, *i*_*k*_+1=*n*+1).

Talking about permutation and combination, division has S ways in total:

(2) $$\begin{array}{c} S=C_{n-1}^{k-1}=\frac{\left(n-1\right)}{\left(n-k\right)!\left(k-1\right)!}. \end{array}$$

Among these ways, one or several must be more optimal with the smallest sum of squares of the total deviations of each classification.

If there are *n* ordered samples, each one is an *m*-dimensional vector, then the correlation matrix *X* can be built:

(3) $$\begin{array}{c} X=\begin{array}{ccc} x_{11} & \ldots & x_{lm}\\ \vdots & & \vdots \\ x_{nl} & \ldots & x_{nm} \end{array}. \end{array}$$

If the dimensions of characteristic values of indicators are different, it is necessary to perform nondimensionlessization with the following formula:

(4) $$\begin{array}{c} x_{ij}^{\prime }=\frac{x_{ij}}{x_{\max j}}. \end{array}$$

*x*_*ij*_′ is the characteristic value after nondimensionlessization and *x*_max,*j*_ is the maximum in the column of the *j* indicator.

Suppose *P* class contains samples {*x*(*i*),  *x*(*i*+1),…,  *x*(*j*)}(*j* > *x*) recorded as *P*={*i*, *i*+1,…, *j*}. Mean of the class is

(5) $$\begin{array}{c} \bar{xp}\;=\frac{1}{j-i+1}\sum_{i=1}^{j}x\left(t\right). \end{array}$$

*D*(*i*,  *j*) is used to indicate the diameter of class, and it can be recorded as

(6) $$\begin{array}{c} D\left(i,j\right)=\sum_{t=i}^{j}{\left(x\left(t\right)-\bar{xp}\right)}^{T}\left(x\left(t\right)-\bar{xp}\right). \end{array}$$

Essence of defining the optimal segmentation of objective function is to find a certain set of points that the sum of squares of the total dispersion of each classification is the smallest. Thus, the objective function is defined as

(7) $$\begin{array}{c} B\left(n,k\right)=\sum_{t=1}^{k}D\left(i_{t},i_{t+1}-1\right). \end{array}$$

The smaller the objective function value, the smaller the internal difference and the larger the differences among classes. Segmentation that minimizes the value of the objective function is the optimal one, i.e.,

(8) $$\begin{array}{c} B^{*}\left(n,k\right)=\min\sum_{t=1}^{k}D\left(i_{t},i_{t+1}-1\right). \end{array}$$

*B*(*n*, *k*) is the optimal *k* segment of *n* ordered samples. The theorem is as follows: the optimal *k* segment of ordered samples series {*x*1,  *x*2,…, *xn*} must be completed by adding a segment after the optimal *k-1* segment *B*(*n*, *k* − 1) of one of its truncated sections. Therefore, the optimal two-division error formula can be obtained:

(9) $$\begin{array}{c} B^{*}\left(n,2\right)=\underset{2\leq i\leq n}{\min}\left\{D\left(1,i-1\right)+D\left(i,n\right)\right\}. \end{array}$$

Then, the recursion formula of the optimal *k* segment is obtained:

(10) $$\begin{array}{c} B^{*}\left(n,k\right)=\underset{k\leq i\leq n}{\min}\left\{B\left(i-1,k-1\right)+D\left(i,n\right)\right\}. \end{array}$$

If the number of *k*(1 < *k* < *n*) is known, the segmentation method that minimizes *B*(*n*, *k*) is as follows.

Find the segment point *i*_*k*_ at first so that *B*^*∗*^(*n*,  *k*) is minimized, i.e.,

(11) $$\begin{array}{c} B^{*}\left(n,k\right)=B^{*}\left(ik-1,k-1\right)+D\left(ik,n\right). \end{array}$$

So, the *k* class is *Pk*={*ik*,  *ik*+1,…,  *n* }. Then, search for *i*_*k*_ − 1, making it

(12) $$\begin{array}{c} B^{*}\left(i_{k}-1,k-1\right)=B^{*}\left(i_{k-1}-1,k-2\right)+D\left(i_{k-1},i_{k}-1\right). \end{array}$$

Thus, we obtained *k*-1 class as *P*_*k*−1_={*i*,  *i*_*k*−1_+1,…, *i*_*k*_ − 1}.

The rest may be deduced by analogy, all the classifications *P*_1_, *P*_2_,…, *P*_*k*_ can be obtained, which is the classification result of the optimal *k* classification. This is the result of the optimal *k* classification. Then, the curve of objective function with the number of *k* segment is plotted, and the *k* value corresponding to the turning point of the curve is the optimal segmentation number. Calculate the absolute value of the slope of the curve at each segmentation point:

(13) $$\begin{array}{c} f\left(k\right)=\left|\frac{B^{*}\left(n,k\right)-B^{*}\left(n,k-1\right)}{k-\left(k-1\right)}\right|. \end{array}$$

Draw the *f*(*k*)*-k* curve. The larger *f*(*k*) is, the better the *k* classification is than the *k*−1 segmentation. When *f*(*k*) is close to 0, there is no need to continue the subdivision. Generally, *k* that corresponds to the maximum of *f*(*k*) is taken as the most optimal number of classifications.

CLSI (Clinical Laboratory Standard Institute) EP28-A3c [12] guideline does not set inclusion and exclusion criteria for reference individuals. This study refers to standard document for the Chinese adult reference interval [13]. Finally, children aged 1–<7 years old from communities and health centers as well as adolescents aged 7–<18 years old from primary schools, junior middle schools, and high schools in Jilin Province, who were apparently healthy, were targeted.

Selection process could be through into 3 steps: (1) questionnaire; (2) physician evaluation; and (3) laboratory screening. Experiment personnel issued the questionnaires at designated institutions and required guardians to fill it out strictly according to the facts, including height, weight, diet, health status, family history, medical conditions, diseases, recent infection, history of surgery within 6 months, blood donation or transfusion within 4 months, and pharmacy history within 2 weeks.

Questionnaires were collected and reviewed. There would be a pediatrician assessing health status of subjects at a certain day every week. After that the subjects were informed to draw blood at a designated place every week as required.

Laboratory exclusion criteria were as follows: HBsAg positive, HCV positive, and HIV antibody positive; serum creatinine (male) > 97 *μ*mol/L; serum creatinine (female) > 73 *μ*mol/L; serum uric acid > 475 *μ*mol/L; fasting plasma glucose > 7.0 mmol/L; serum albumin < 35.0 g/L; C reactive protein > 10.0 mg/L; serum creatine kinase > 500 U/L; hemoglobin (male) < 120 g/L; hemoglobin (female) < 110 g/L; and white blood cell count < 3.0 × 10^9^/L, or > 12.0 × 10^9^/L.

This study was approved by the institutional ethics committee of the First Hospital of Jilin University (2016–306). Subject and his/her guardian signed up written consent. All methods/experiments were carried out in accordance with relevant guidelines and regulations (*Declaration of Helsinki*).

EP28-A3c (13) stipulates that there should be no less than 120 reference individuals each partition. Since partition points remain unknown, enrollment and sample collection work lasted continuously from September 2017 to December 2018 to ensure that every 1 year gap and each gender are included over 120 individuals. Recruitment was from 5 administrative regions (9 in total) of Jilin Province.

Subjects were guaranteed regular diet and exercise 3 days ago and fasted for >8 hours (age under 3 was suggested 3–6 hours) before blood collection. Four millimeters of venous blood were collected in plastic vacutainers, placed at room temperature for 30 minutes and then centrifuged at 3,000 rpm for 10 minutes. Hemolyzed, lipemia, or jaundice specimens were removed. Serum in Changchun city should be transported to the First Hospital of Jilin University within 2 hours after separation for analyzed, while serum beyond Changchun city required 8 hours by cold chain trucks.

Ortho VITROS 5600 automatic biochemical analyzer and reagents were applied to detect serum AST (multipoint rate method, reagent containing pyridoxal 5-phosphate), LDH (multipoint rate method), CK (multipoint rate method), and CKMB (multipoint rate method). Calibrators and quality control materials were also supported by Ortho Clinical Diagnostics.

Database was reviewed and miscellaneous data were eliminated. Data processing is carried out based on EP28-A3c (13). Outliers were removed using the Dixon method and re-evaluated by box plots. Kolmogorov–Smirnov test was conducted to determine if the data followed Gaussian distribution. If it was satisfied, percentiles of each index were calculated as *P*5,  *P*10,  *P*15,…, *P*100; if not, calculated after Box–Cox normality transformation with Mintlab. Percentiles were substituted as impact factors into the model, and then an ordered sample matrix *X* with a capacity of 17 and each one being a 20-dimensional vector were built. After obtaining the optimal classification number and points, *Z* test was to verdict whether there was statistical difference between classes. If *Z* > *Z*^*∗*^, classes were combined; otherwise, RI should be established, respectively. Gender difference was performed in the same way. Average or median of each indicator per 1 year old was calculated according to the normality, and a matrix with capacity of 17 and each one being a 4-dimensional vector were built by substituting the calculation into the model. Trends of indicators were assessed by scatter plots, and the partition results were verified.

Statistical analysis and figures were completed using LMS, Excel, Medcalc, SPSS, Matlab, and Mintlab.

## 3. Results

### 3.1. Baseline Information and RIs of Myocardial Enzyme

There were 6,322 healthy children and adolescents enrolled, including 2,998 subjects from Baishan, 2,193 subjects from Changchun, 500 subjects from Songyuan, 466 subjects from Yanbian, and 165 subjects from the city of Jilin. For 3,119 males and 3,203 females, sex ratio was 1 : 1.03. RI was obtained by performing *Z* test after the age segmentation using Fisher's method (Table 1).

**Figure 1:**
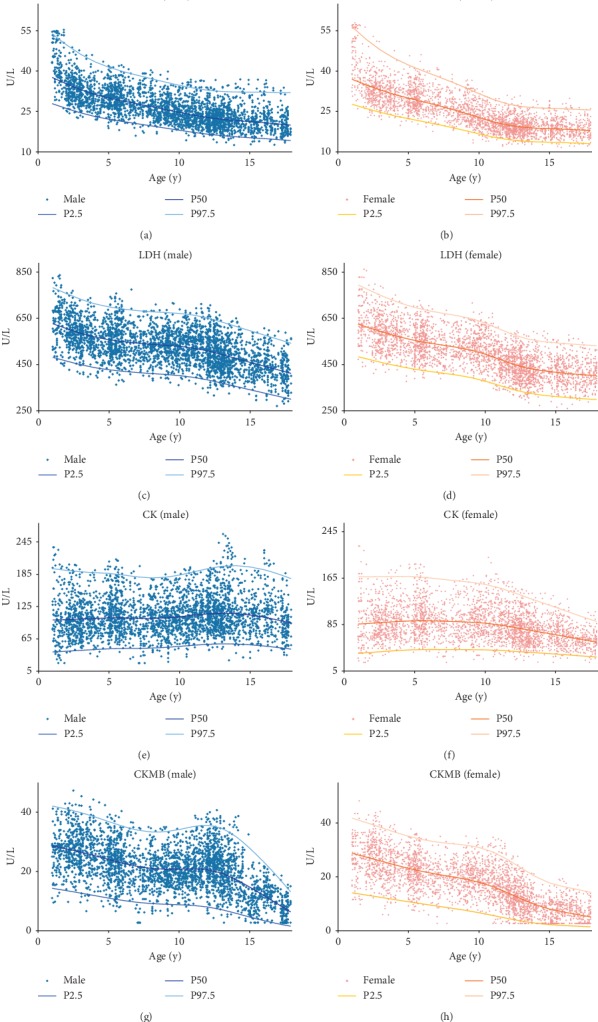
Trends of AST, LDH, CK, and CKMB among healthy population aged 1–<18 years old.

Taking CKMB as an example (including males and females), objective function *B*^*∗*^(*n*, *k*)*-k* and the nonnegative slope *f*(*n*, *k*)*-k* curve were plotted (Figure 1). It was shown that the error function *B*^*∗*^(*n*, *k*)*-k* decreased as the number of segments increased. At *k* = 3, *B*^*∗*^(*n*, *k*)*-k* curve became the steepest and made a bend, with *f*(*n*, *k*)*-k* curve reaching its peak. Hence, the optimal classification number *k* was 3, and specific endpoints were presented by code results. With *B*^*∗*^(*n*, *k*)*-k* and *f*(*n*, *k*)*-k*, the optimal classification numbers *k* for AST, LDH, or CK were also the same as that of CKMB. However, reference interval of CK does not need partition after *Z* test.

### 3.2. Age and Sex Related Trends

**Figure 2:**
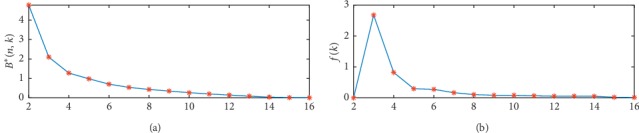
*B* ^*∗*^(*n*,  *k*) − *k* and *f*(*k*) − *k* are applied to determine the optimal segmentation number.

From the scatter plot of each indicator (Figure 2), it was shown that AST decreased and the speed slowed gradually, and males' values overwhelmed females' values. LDH presented disparate descending rate among age partitions among 1∼<4, 4∼<12, and 12∼<18 years old. CK stood quite stable, sharing the same RI in population aged 1–<18 years. CKMB differed between sexes at 6 years age; for male, CKMB remained stable during 6∼<14 years old and began to decline thereafter; for female, CKMB continued to decline with age.

### 3.3. Determination of Cardiac Development in Population Aged 1–<18 Years

Mean or median of each indicator from every year was included in this model; then, cardiac development was partitioned as 1∼<6, 6∼<13, and 13∼<18 years old. The partitions for male and female were calculated as 1∼<6, 6∼<14, and 14∼<18 and 1∼<6, 6∼<12, and 12∼<18 years old, respectively. It can be speculated that adolescent females' heart development started to change earlier.

## 4. Discussion

### 4.1. Status of Pediatric RI Research

Pediatric RI study is the focus of clinical laboratory medicine [13]. Although many details are not quite clear yet, such as reference individual criteria, partition method, and efficacy verification [12], this project is of great significance. There is no feasible pediatric RI in China nowadays. RIs from various sources are applied in clinical laboratories at all levels, such as local databases, manuals, textbooks, or literature, of which data are outdated and credibility is quite doubtful [1]. Therefore, diagnosis of many pediatric diseases relies on rich experience of pediatricians largely, and it is often the case that explanation of results is inconsistent with diagnosis due to improper RIs. Establishment of an accurate and reasonable pediatric RI is critical for monitor and treatment of illness.

Many indicators change constantly due to children and adolescents' growth and development [14, 15]. For example, alkaline phosphatase in neonates is slightly higher than adult level within 3 months after birth and turns out to be 2-3 times higher during puberty [16]. N-terminal B-type natriuretic peptide concentration in pediatric population reached 260% of adults' [17]. It is obvious that simply following adult RI is likely to cause disturbance in some situations, which seriously affects clinical decision-making. In addition, the whole process of variation is continuous but staged owing to time-phased hormones and external stimuli [14]. Summing up the above, underlying idea of EP28-A3c [12] is “Let data speak”, leading to investigation of how to properly present and apply the information data contains. There is a still lot of work to do on this field.

### 4.2. Fisher's Optimal Segmentation

The goal of RI research is to make it a concise, effective, and practical diagnostic tool. Age is an ordered variable apparently. For instance, partitions of indicators as AST and LDH that steadily rise or decline are judged from scatter plot subjectively. Changing point refers to a certain moment when the sudden alternation of an ordered sequence happens. Fisher's optimal segmentation includes multiple percentiles from massive data that reflect characteristics of totality as impact factors into the model. Fisher's optimal segmentation method is used as a clustering method for ordered samples. It splits a sequence of ordered samples according to principle that distinguishes among partitions being the most distinct as well as internal differences being smallest. The optimal solution is to minimize the sum of the squares of the total dispersion into groups, while all possible classifications maintain the time continuity. It has no strict requirements of data form with selection of percentiles being less affected by extreme values although certain information is lost.

The results are in agreement with some clinical cardiovascular studies. As for AST and LDH, the downward trends might be due to increased liver size, muscle mass, and fat-muscle distribution changing with age [18]; CK and CKMB are greatly affected by physical activities. We found the partitions are quite consistent with phases of elementary school (6–12 years old), junior middle school (12–15 years old), and high school (15–18 years old) in China, when physical education and sports in the peer group could exert a great impact.

Combination of multimarkers is commonly applied to assess myocardial damage. Trend of percentiles 75th, 90th, 95th, and 99th of LVMI ^2.7^ (body height to the allometric power of 2.7) increases gradually over 10–12 years old, stating that a fixed cut-off point would be theoretically inappropriate and generates a pattern of age-dependent subdiagnosis [19]. We had found that cardiac development was partitioned at 11 years of age in females as well as 12 years of age in males. Khoury et al [20] states that end-diastolic and end-systolic volumes of both ventricles indexed to weight exhibits a slight decrease from childhood to adolescence where a plateau shows at around 14 years old among 99 subjects of 8–20 years old, close to our results that cardiac development partitioned at 12 years old or so. Recent work has used steady-state free-processing (cine-SSFP) protocols to yield pediatric cardiac reference values in 60 children, divided into age groups of 8–11 years, 12–14 years, and 15–17 years old [21], which is different from our results for preadolescent period as 1–5 years and 6–12 years.

Though only being significant in the older group, sex differences are noted in both age groups when analyzing pediatric patients (8–15 years) versus adolescents/young adults (16–20 years) [22]. Besides, data shows that females have a lower prevalence of left ventricular hypertrophy (LVH) than men under any given level of blood pressure [23]. It might be the reason that most of the indicators begin to present higher levels in males in puberty. Goble et al. reports that body size, and in particular lean body mass, explains much of the variability in cardiac growth seen in children [24]. Thus, late bloomer as boys may not start to appear cardiac growth until fully growing stages as adolescence.

## 5. Limitation

First, this model cannot include age and sex into the model at the same time, as seen when the 4 indicators were combined to reflect cardiac development. Hence, it is not quite accurate for identifying the exact age when sex difference begins to show. Second, results of this study are a general trend to determine the optimal segmentation of cardiac development in children and adolescents aged 1∼<18 years old. Partition may vary slightly if age coverage changes.

## 6. Conclusion

Fisher's optimal segmentation method was used to establish RIs for myocardial enzyme activity in healthy children and adolescents aged 1–<18 years in Jilin Province, China. To describe cardiac development process, multiple percentiles were selected as impact factors included in the model for partition investigation. The method presented multidimensionality, continuity, and loop calculating as dealing with such problem. However, it also has some shortcomings such as inability to assess both age and sex at the same time. In future research, impact of partition on the overall interval should be delved into.

## Figures and Tables

**Table 1:** Age- and sex-specific reference intervals of myocardial enzyme activity in healthy population aged 1∼<18 years (*n* = 6322).

Analytes	Age partition	Sex partition	*n*	LL	UL	Confidence interval for LL	Confidence interval for UL
AST, U/L	1	M + F	263	29.1	57.2	26.2–29.7	56.4–57.2
	2∼<10	M + F	2928	20.1	40.7	19.9–20.4	40.4–41.1
	10∼<18	M	1518	15.7	33.8	15.4–16.1	33.4–34.1
		F	1613	14	27.7	13.5–14.3	27.3–28.8
LDH, U/L	1∼<4	M + F	1115	461	761	456–466	753–769
	4∼<12	M + F	2910	397	674	393–400	670–678
	12∼<18	M	1119	339	624	334–344	617–632
		F	1178	316	555	312–320	549–561
CK, U/L	1∼<18	M + F	6322	40.2	179	39.5–40.9	177.1–181
CKMB, U/L	1∼<6	M + F	1976	11.7	39.1	11.3–12.1	38.7–39.6
	6∼<14	M	1642	8.9	34.2	8.5–9.3	33.7–34.7
		F	1629	4.5	30.2	4.1–4.8	29.7–30.7
	14∼<18	M	507	2.5	26.3	2.2–2.9	25–27.6
		F	568	2.7^*∗*^	15.1	2.7^*∗*^	14.4–16.7

^*∗*^2.7U/L is the lowest detection limit. Actual values could be lower.

## Data Availability

The testing data used to support the findings of this study are available from the corresponding author upon request.
